# Potential Synergies between Nature-Based Tourism and Sustainable Use of Marine Resources: Insights from Dive Tourism in Territorial User Rights for Fisheries in Chile

**DOI:** 10.1371/journal.pone.0148862

**Published:** 2016-03-29

**Authors:** Duan Biggs, Francisca Amar, Abel Valdebenito, Stefan Gelcich

**Affiliations:** 1 ARC Centre of Excellence for Environmental Decisions, Centre for Biodiversity & Conservation Science, University of Queensland, Brisbane, Queensland 4072, Australia; 2 Department of Conservation Ecology and Entomology, Stellenbosch University, Private Bag X1, Matieland 7602, South Africa; 3 Center of Applied Ecology and Sustainability (CAPES) & Centro de Conservación Marina, Departamento de Ecologia, Facultad de Ciencias Biológicas, Pontificia Universidad Católica de Chile, Santiago, Chile; 4 Laboratorio Internacional en Cambio Global, Consejo Superior de Investigaciones Cientificas, Instituto Mediterraneo de Estudios Avanzados, Esporles, Mallorca, Spain; 5 Bren School of environmental science and management, University of California Santa Barbara, Santa Barbara, California 93106, United States of America; U.S. Geological Survey, UNITED STATES

## Abstract

Novel solutions to conserve biodiversity whilst allowing for resource harvesting are urgently needed. In marine systems, Territorial User Rights for Fisheries (TURFs) are promoted to enable sustainable use of resources. We investigate the potential for synergies between nature-based tourism and TURFs on Chile’s central coast. Of 135 recreational divers surveyed, 77% indicated that the fish species they preferred sighting were declining and 80% indicated that they would dive more often in TURFs, which have higher abundance of favoured species. Regression analysis shows that respondents that perceive that TURFs fulfil a conservation function are more willing to pay to dive in a TURF. However, respondents who understand the bureaucratic functioning of a TURF are less willing to pay, and there is diversity in how divers feel payments should be made. A participatory approach is required to navigate these complexities to achieve synergies between nature-based tourism and resource harvesting in TURFs.

## Introduction

The biodiversity crisis is worsening amidst increasing pressure on natural resources. Innovative strategies to conserve biodiversity, whilst allowing for resource use by local communities are urgently needed [1, 2, 3]. In marine systems, territorial user rights for fisheries (TURFS) have been promoted over the past decade as an instrument which can enable the sustainable utilisation of marine resources by providing appropriate access rights and incentives [4, 5]. At the same time, in both marine and terrestrial systems, nature-based tourism has been widely promoted as a way to achieve both economic gains and conservation [6–9].

Nature-based tourism in marine environments has typically centred around coral reefs, or iconic marine species such as whales and sharks [10, 11]. Marine tourism not associated with iconic mammals or coral reefs receives less attention but nevertheless, could play an important socio-economic role in temperate ecosystems (e.g. [12]). If the marine diversity and abundance is higher in TURFs than outside of TURFs and if recreational divers have a preference for higher diversity and abundance, the potential for synergies between nature-based tourism and resource extraction in TURFs exists.

Chile, has a national TURF policy following the 1991 Fishery and Aquaculture Law [13]. Currently, there are over 800 TURFs in Chile along 2500km of coastline [2, 13]. To be granted a TURF, community artisanal fisher associations (hereafter associations) must undertake a baseline study of their TURF and develop management plans with the aid of technical assistance that need to be approved by the undersecretary of fisheries. These fisher associations are responsible for the surveillance and enforcement with support from the National Fisheries Service [14]. TURF management plans consider economically valuable benthic species, and it is forbidden to extract benthic species not considered in the management plan. Recently, the biodiversity conservation implications of TURFs began to be assessed scientifically [2]. Results of these studies showed that TURFs in kelp forests can sustain significantly higher reef fish and macroinvertebrate species richness, biomass, and density compared with kelp forests in open-access areas which have the same habitat complexity characteristics [15, 16]. Furthermore, results show that TURFs with different enforcement levels had significant differences in macroinvertebrate species richness, density, and biomass, suggesting that the level of enforcement, aimed at preventing poaching in TURFs, is associated with biological diversity [2, 17]. These findings from different studies [2, 15, 17, 18] suggest that beyond possible consequences of site-selection bias, species diversity patterns within TURFs are driven, to some extent, by enforcement and management over time. In this sense, well-enforced TURFs (i.e. those with surveillance agreements in place) have been proposed to play an important role as ancillary marine conservation instruments [19, 20].

In addition, Chile also has a growing nature-based tourism industry (SERNATUR 2012). However, to date, the potential synergies between TURFS and nature-based marine tourism have not been investigated. Presently, there are no clear regulations to support or prohibit recreational diving in TURFs, and there is uncertainty among divers about access rules and procedures. Our paper addresses this gap by investigating the potential benefit of integrating recreational diving in the management and income generation strategies of TURFs. To our knowledge, this is the first study that empirically examines the potential for synergies between extractive use, recreational use, and conservation within TURFs.

Specifically, our study aimed to address the following questions: 1) what is the perception among recreational divers of the changing state of marine life, and whether this is related to diver experience; 2) which marine species do recreational divers prefer to observe; 3) what is the perception of TURFs among divers, and are they willing to pay a user fee to access TURFs with greater biodiversity and abundance; and finally 4) which variables predict the willingness of recreational divers to pay a visitor’s fee to dive in TURFs.

## Materials and Methods

### The study area

**Fig 1:**
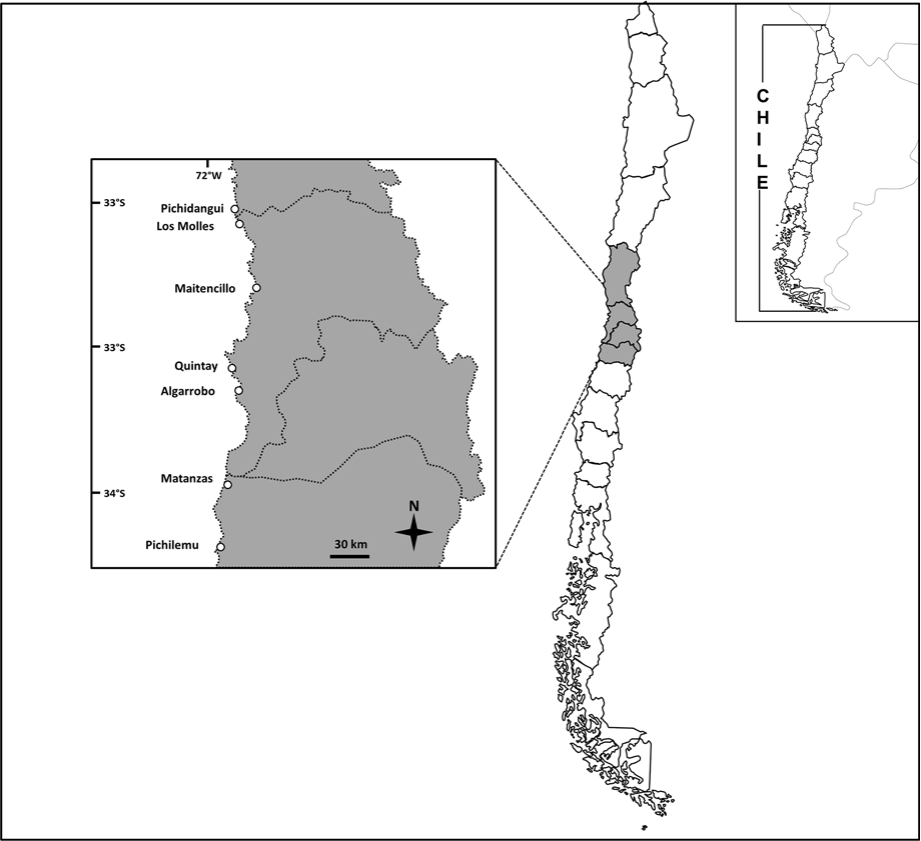
Map of the study area.

Currently in Chile there are around 800 TURFs legally allocated to fisher organizations [21] out of which around 450 are fully functioning [22]. Although there is heterogeneity in their performance, they account for more than 1,100 km2 of the nearshore seascape, with an average size of approximately 100 hectares and an average distance between them of 4–10 km [13]. The seascape between TURFs are de facto open access areas. TURFs are created and assessed to manage economically important benthic species such as the carnivorous muricid gastropod Concholepas concholepas (managed in 80% of TURFs), key-hole limpets, *Fissurella* spp. (70%), and the red sea urchin *Loxechinus albus* (30%) and more recently for algae species (Castilla et al. 1998; Castilla et al. 2007). Diving for benthic resources within the TURF is usually restricted to a few times a month [13]. In fact, the average income for fisher associations which comes from resources from within the TURFs is around 20% [22]. Under these circumstances, the potential for developing tourism within TURFs and establish synergies between TURFs used for extracting resources and as a tourist destination is possible. To explore these possible synergies, research surveyed recreational divers who usually dive in central Chile between Pichidangui (32°08′00″S 71°32′00″W) and Pichilemu (34° 23′ 0″ S, 72° 0′ 0″ W), where there are currently around 80 TURFs granted to fisher associations and where recreational diving has experienced an important growth in the past ten years (Fig 1).

### Surveys

We surveyed 135 recreational scuba divers using an online survey (es.surveymonkey.org). The mailing list of divers used to contact respondents was provided by active dive centres located in central Chile. We contacted 180 divers and 150 responded that they were willing to participate in the survey. Of the 150 surveys submitted we received 135 completed surveys. We pre-tested the survey tool with 25 recreational divers. Fisheries scientists and marine biologists were explicitly excluded from the survey in the invitation letter and through a screening question. Recreational scuba divers do not practice spear-fishing in Chile, and recreational free-divers and artisanal spear-fishers were excluded from the dataset. Data were collected between July and December 2008 and questionnaires were administered in Spanish. The study and informed consent was approved by the ethical commission of the Faculty of Biological Sciences at the Pontificia Universidad Católica de Chile for FONDECYT project 11070034. Respondents were informed about the objectives and dissemination of the study results. The study complies with the FONDECYT ethical codes.

We collected information on a range of socio-economic variables (age, income, occupation, education levels) that are known to influence willingness to pay user fees or contribute to conservation outcomes [23–25]. Moreover, awareness of changes to ecological conditions and broader conservation attitudes may affect willingness to pay [26, 27]. Attitude towards and knowledge of marine conservation was assessed through a number of questions (Table 1). Five-point Likert scales with anchor points were used to assess whether respondents think that marine conservation is important, and whether it is important to have marine reserves in Chile; 1 = very important to 5 = very unimportant [28]. We used a binary variable to assess whether respondents know of a marine protected area in Chile. In addition, we evaluated perceptions of changes in the abundance of fish and invertebrates using 5 point Likert scales.

**Table 1:** Descriptive Statistics.

Variable	Mean	Median	Std Dev	Min	Max
Do you know of a species that is in danger of extinction?*	0.820	1	0.384	0	1
Have you, or do you participate in a conservation organisation?*	0.290	0	0.455	0	1
Do you think it is important to have marine reserves in Chile? **	4.900	5	0.445	1	5
Do you think TURFs fulfil a conservation role?**	3.640	4	1.406	1	5
Do you think that marine conservation is important?*	4.689	5	0.384	1	5
Do you understand how TURFs operate in terms of structures and bureaucracy?*	0.56	1	0.499	0	1
Have you observed a decrease in the abundance of fish species that you like to see during your lifetime?***	4.08	4	0.820	2	5
Have you observed a decrease in the abundance of species of invertebrates that you like to see during your lifetime?***	3.7	4	0.955	1	5
Have you had problems entering a TURF to go diving before?^α^	2.90	3	1.154	1	4

* = binary variables (0 = no; 1 = yes)

** = measured on a 5 point Likert scale (1 = not important to 5 = very important)

*** (1 = large increase; 3 = no change; 5 = large decrease)

^α^(1 = never, 2 = once, 3 = yes, a few times, 4 = yes many times)

n = 135.

Diver’s knowledge of how TURFs function and the interaction between the dive experience and the abundance and diversity of marine life in TURFs compared to open access areas was evaluated through a number of questions. Divers were asked to respond to the statements, ‘when I see more fish, diving is better’; ‘I see more species in TURFs than in open access areas’; ‘the same species that I see in open access areas grow to a larger size in TURFs’; and ‘the same species that I see in open access areas occur in greater abundance in TURFs on a 5 point Likert scale (1 = strongly disagree to 5 = strongly agree). In addition, the marine species that recreational divers prefer to observe was assessed by asking open-ended questions in which respondents indicated which three marine fish and invertebrates they most like to observe. Divers were asked whether or not they think TURFs fulfil a conservation role on a 5 point Likert scale (1 = insignificant role to 5 = a very important role).

Respondents who are aware of TURFs and their bureaucratic and administrative functioning may be more or less likely to be willing to pay to access TURFs for recreational diving. Whilst there is an increasing willingness to pay entry fees to access marine reserves, a lack of trust in the collection agency and how the money will be spent deters the willingness to pay [29]. Thus, respondents who do not trust the way income to TURFs will be managed, and may feel that they are characterised by excessive bureaucracy and red tape, superfluous administrative procedures, and do not trust money will be spent efficiently are likely to be less inclined to want to pay to access these sites for diving [30, 31]. Respondents that trust that TURFs are administrated in an efficient fashion without superfluous bureaucracy are more likely to be willing to pay additional charges to access these sites for diving. We measured whether or not recreational divers understood how TURFs function in terms of structure and bureaucracy (binary variable), and whether they felt TURFs fulfilled a conservation function (measured on a 5 point Likert scale). In addition, we collected data on the characteristics of trips undertaken by recreational divers including the number of hours normally travelled to a diving site. We used a four-point scale on whether or not divers had experienced problems trying to dive in TURFs before (Table 1).

Finally, as the response variable for the willingness to pay analysis, respondents were asked how much they would be willing to pay to dive in a well enforced TURF with greater diversity and abundance of commercial and non-commercially valuable marine reef-fish species and invertebrates. The diversity and abundance data of fish and invertebrates used in the survey came from empirical surveys and photographs of TURFs in central Chile [15]. Respondents could choose that they were not willing to pay at all, that they would pay up to US$6 or that they would be willing to pay more than US$6. A US$6 fee was used, as this is the median price for an entry fee for a natural protected area in the national system for protected areas of Chile [32]. In addition, respondents were asked whether payments should be made directly to associations or whether it should be managed by a third party such as a foundation or an NGO. In addition the survey asked whether such a payment should be a voluntary donation only.

### Analysis

We assessed whether perceptions of the changing state of marine life was related to diver experience using Kendall tau’s correlation coefficient. The Kendall tau coefficient is appropriate for many tied ranks, which we have in our dataset [33]. A stepwise forward multinomial logistic regression was performed using IBM SPSS version 20 to determine which variables predict the respondent’s willingness to pay [33]. Multinomial logistic regression is used when the dependent variable is categorical and the explanatory variables are continuous or categorical, and it is used to determine the variables that predict respondent willingness to pay [34–36]. Responses to the statement whether TURFs fulfil a conservation role, do you think that marine conservation is important, and do you think it is important to have marine reserves in Chile were collapsed from five categories into two for the analysis. This was necessary because some categories in the 5 point scale had very few entries. In the analysis, respondents not willing to pay was used as the reference category.

## Results

**Fig 2:**
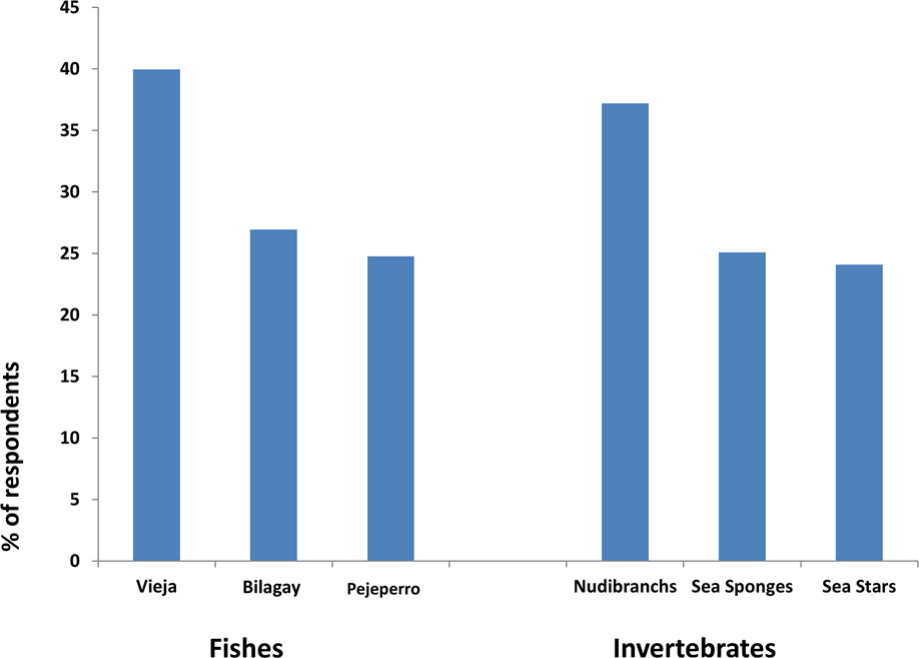
The species and taxonomic groups indicated by respondents in response to the questions: which fish and invertebrates do you most like to observe when you go diving on the coast of central Chile.

The average age of divers was 32 (range 16–78). Respondents had a high level of education, 30% had completed post-graduate degrees, a further 48% had completed pre-graduate studies and 15% technical studies. Only 9% of respondents had been diving for less than a year, 33% for between one and four years, 23% for between five and nine years, and 27% for between ten and 19 years. Only 8% of respondents had been diving for more than 20 years. Divers perceived that the abundance of fish that they like to see had decreased by 76% and invertebrates by 60%. Divers perception of decreasing abundance of fish (p < 0.01) and invertebrates (p < 0.05) was positively correlated with diver experience. The three most frequently reported fish species that respondents preferred to observe were the ‘Vieja’ *Graus nigra* (40%), ‘Bilagay’ *Cheilodactylus variegatus* (27%), and the ‘Pejeperro’ *Semicossyphus darwinii* (25%) (Fig 2). The three most desired invertebrates for observing were nudibranchs (37%), sea sponges (25%) and sea stars (24%) (two species dominate on the coast of central Chile *Stichaster striatus* and *Meyenaster gelatinosus*) (Fig 2).

**Fig 3:**
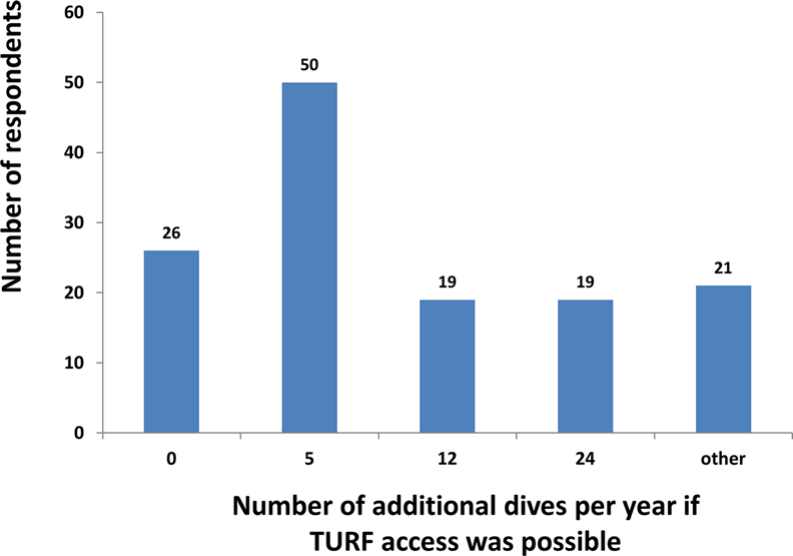
The number of respondents who indicated with which frequency they would go diving more often per year in TURFs that have greater diversity and abundance of fish and invertebrates if there was a clear policy TURF dive access policy. N = 135.

There was extensive variation in the attitude towards, and the awareness of, conservation as well as in the knowledge of TURFs (Table 1). The majority of respondents knew of a species that is in danger of extinction, whereas only 29% had historically been, or were members of a conservation organisation at the time of the survey. There was virtually unanimous agreement about the need to have marine reserves in Chile. There was a high level of agreement about the importance of marine conservation in Chile, and 56% of respondents indicated that they understand how TURFs operate in terms of structure and bureaucracy (Table 1). Overall 62% of respondents feel that TURFs fulfil an important or very important conservation role. Finally, the majority of respondents felt that when they see more fish diving is better (Table 1). In addition, the majority of respondents felt that there is a greater diversity of species, greater number of larger individuals, and a greater abundance of individuals in TURFs than in open access areas (Table 1). Fifty-eight percent of respondents have had problems in attempting to access TURFs to go diving in the past. Eighty percent of divers surveyed indicated that they would go diving more often if there was a clear regulation that allowed access to diving sites in TURFs where there is higher diversity and abundance of fish and invertebrates (Fig 3). In addition, 65% of respondents indicated that they would go diving at least five times more often if a policy enabling access to TURFS were in place.

### Willingness to pay a visitor fee to access diving sites inside TURFs

Seventy-six percent of divers indicated that they would be willing to pay to dive inside TURFs where there is more biodiversity and abundance. However there was substantial variation in how those that indicated they were willing to pay felt that payments or contributions to the management costs of TURFs should be made. Thirty-eight percent of respondents indicated that they were happy to pay an entrance fee to access TURFs, and 29% felt that payment should be made to and managed by a third party.

Thirty two percent of respondents were not willing to pay to enter into TURFs to dive, whereas 47% were willing to pay US$6 or less, and 21% were willing to pay more than US$6. However, there was substantial variation in how those that indicated that they were willing to pay felt that payments or contributions to the management costs of TURFs should be made. The results of the regression analysis showed that respondents who felt that TURFs fulfil a conservation role were positively related to willingness to pay US$6 or more to enter to dive in TURFs (Table 2). However, respondents who were aware of the bureaucracy and the structures through which TURFs function were inversely related to willingness to pay (Table 2).

**Table 2:** Results of multinomial logistic regression with willingness to pay as the dependent variable. Not willing to pay was used as the reference category. Pseudo R^2^ = 0.118 (Cox and Snell), 0.134 (Nagelkerke). Model Chi Squared (*df* = 4) = 17.008, *p* = 0.02.

			95% CI for Odds Ratio		
		B (SE)	Lower	Odds Ratio	Upper
Willing to pay up to US$6	Intercept	0.350(0.420)			
	I understand how TURFS operate	-1.023(0.435)*	0.153	0.360	0.844
	I think that TURFS fulfil a conservation role	1.005(0.423)*	1.192	2.732	6.264
Willing to pay more than US$6	Intercept	-0.536(0.525)			
	I understand how TURFS operate	-1.143*	0.118	0.118	0.865
	I think that TURFS fulfil a conservation role	1.387*	1.402	4.003	11.427

*p <0.05.

## Discussion

Our paper shows that recreational divers perceive that the diversity and abundance of fish and invertebrates are declining, and the level of experience of divers was positively correlated with perceived decline. Findings from elsewhere suggest that more experienced, and specialist divers are indeed more likely to be aware of changes in ecological condition [37, 38]. In addition, these nature-based tourists would prefer to dive where there are higher levels of diversity and higher abundance of fish and invertebrates, that well-enforced TURFs can provide [2]. Our results show that the majority of recreational divers are willing to contribute to the management costs of TURFs through paying an access fee. Thus, there is potential to synergise income from nature-based tourism with sustainable use on Chile’s central coast. However, our results suggest that the lack of trust in the efficient functioning of the administration of a TURF and how the entry fee to TURFs may be managed and used presents a challenge to harnessing implementing an entry fee system.

### Perceptions of decline and preferences to dive in TURFs

Respondents perceive fish and invertebrates are in decline, this aligns with scientific findings of the state of coastal Chile’s marine ecosystems [39, 40]. Respondents prefer to dive where there is higher diversity and higher abundance of large fish and invertebrates. The majority of respondents indicated they would dive more often and that they are willing to pay to enter areas with higher abundance and diversity. Our findings align with studies in tropical coral reef environments where higher levels of coral and fish abundance and diversity, and reef structural complexity are valued by recreational divers in the Carribean island of Bonaire [41, 42].

The preferences of recreational divers suggest that there is an opportunity to augment income to TURFs through nature-based tourism. In addition to species richness, biomass, and density of macro-invertebrates and fish, the densities of the desired sea stars are higher in well-enforced TURFs than in poorly enforced TURFs [2]. Moreover, in TURFs with high levels of enforcement, six species of fish including the favoured ‘Bilagay’ and ‘Vieja’ had similar densities to a well-enforced benchmark no take marine protected area (MPA). Therefore, TURFs with high levels of enforcement can offer a similar experience to no take MPAs with respect to the diversity and abundance of a number of desired taxa.

### TURFs, conservation, and diver’s willingness to pay

Our finding that belief in the importance of conservation is positively associated with willingness to pay an access fee aligns with findings from other studies [26] as well as studies on the monetary value attached to, and willingness to pay for environmental goods [43, 44]. In addition, belief in the importance of conservation predicts the willingness of marine tourism operators to contribute to conservation, including financial contributions [45, 46]. Engagement in co-management through TURFs has changed fishers environmental attitudes with time [16]. Results from elsewhere suggest that nature-based tourism presents a good vehicle through which attitudes towards conservation can be improved [9, 45, 47]. Thus, the development of nature based tourism operations in TURFs could further advance conservation awareness among fishers because fishers will see the financial benefits from having higher levels of biodiversity that divers are willing to pay to see.

An understanding of how TURFs operate in terms of their structure and bureaucracy was inversely related to the willingness to pay to enter to dive in TURFs. Although this result may seem counterintuitive, this finding is supported by results from other studies on willingness to pay for recreation opportunities when the level of trust in the collection agency, and a belief that money will be spent efficiently to achieve conservation outcomes is considered [29, 48]. In the Phillipines, Environmental NGOs were preferred as recipient and management organisations of user fees, above local fishing communities, and local and national government [48]. Similarly, recreational divers that are familiar with management structures and processes in TURFs, may feel that they are overly cumbersome or inefficient.

Our results signal that building trust and strengthening communication between fishers and recreational divers is a challenge that should be addressed in the near future if the synergy between recreational divers and fishers in TURFs is to be successful in Chile. This communication must also consider design choices regarding possible interference between fishing and tourism activities, and ensure that trust between recreational divers and fishers is built. Indeed, in the Community Areas Management Programme for Indigenous Resources (CAMPFIRE) in Zimbabwe, the presence of clear lines of communication between community user groups, local government and recreational hunting companies enabled trust between stakeholders, and was key to the successful development of CAMPFIRE [49, 50].

Importantly, obtaining an estimate of the total potential contribution of nature-based tourism to the management costs of TURFs was not our intention in this study. Willingness to pay analyses are often used to estimate the total potential contribution of nature-based tourism to conservation costs (e.g. [30, 48, 51]). However, our aim was to explore whether recreational divers would in principle be willing to pay an entry to TURFs and which variables predict that willingness. Our results are consistent with other studies assessing willingness to pay in Chilean MPAs [30]. Our results show that 21% of the sampled divers are willing to pay more than six dollars for accessing TURFs, while Gelcich and others [30] show that 20% of nature based tourists would pay more than US$6 to visit the Lafcken Mapu Lahual MPA in southern Chile. This study provides information on the value TURFs could have for recreational divers and the types of social and institutional considerations that need to be accounted for, if a scheme whereby divers pay to access TURFs is established. A further study will be required to obtain an accurate estimate of the total potential contributing of recreational divers to the management costs of TURFs in Chile.

### Complexities of collecting and managing visitor fees

The reticence of recreational divers who understand how TURFs function to pay entrance fees and the heterogeneity of TURF associations in central Chile, suggests that a diversity of approaches are required to successfully design a system for recreational divers access and fee collection. TURF associations vary with respect to their assets, access to credits and existing infrastructure [52], as well as different habitat types they occupy.

Moreover, there is wide variation in income streams to TURF associations. Some have transitioned to tourism-driven forms of income and have systems in place for tourists to pay for services such as fishing access, beach services and others). Other associations are still fully dependent on fishing operations as a source of income as they are distant from large populations of potential tourists, and/or there are infrastructure constraints in accessing dive sites. Different associations have varying levels of capacity to manage the collection of visitor fees, and the trust of divers in each association to manage fees efficiently will differ. For example, in the Maitencillo TURF, beach tourism is already an important source of income and there are fee collection and corresponding management processes in place (M. Herrera, secretary of Maitencillo Association, pers. Comm). TURFs like Maitencillo are therefore more likely to be open to allowing divers to access their TURF for diving purposes, than a TURF association that has never had an experience with any form of tourism. Similarly divers are more likely to have confidence in the way dive access fees are managed, if they perceive that TURF associations have experience managing such fees and are well governed. Indeed, the history of co-management in Chile demonstrates the importance of accounting for local variations, and long-standing institutional practices in the establishment of new management systems [53].

An over-arching coordinating organisation can often play the role as mediator between the tourist and the community groups responsible for delivering a nature-based experience and managing a resource [54]. If such an organisation is established in a way that has the trust of the nature-based tourists visiting these resources, and has an explicit conservation aim, such an organisation may hold a greater level of trust among recreational divers, and they may be more willing to pay to access TURFs [29, 48]. To explore the potential of these different alternatives we advocate for further research aimed at developing a customer driven design of such a brokering system. This research should aim at understanding the key issues which are important for both fisher associations and the demand from tourists and include these aspects in the design of the business model which aims to promote tourism within TURFs.

## Conclusion

Our paper shows that the preferences of recreational divers to see a greater diversity of fish and invertebrates can be satisfied by gaining access to well enforced TURFs and that they are willing to contribute through an entrance fee to the management costs of TURFs. The design and implementation of such a system will need to account for the diversity of local circumstances in central Chile and needs to address the issue of trust. Our findings suggest that there is potential to synergise nature-based tourism, and sustainable utilisation in marine environments, but that to successfully harness this potential, careful consideration of the local social context will be needed.

## Supporting Information

S1 Dataset Dataset for this study, with identifying information removed. (XLSX)

S1 Survey Tool The survey tool used for this study. (PDF)
