# Supplementary material for: Potential Synergies between Nature-Based Tourism and Sustainable Use of Marine Resources: Insights from Dive Tourism in Territorial User Rights for Fisheries in Chile
Source: PLoS One. 2016 Mar 29;11(3):e0148862. doi: 10.1371/journal.pone.0148862 (PMC4811548; doi:10.1371/journal.pone.0148862)
Supplement: S1 Survey Tool — (PDF) [file pone.0148862.s002.pdf]

PONTIFICIA
UNIVERSIDAD
CATÓLICA
DE CHILE

Consentimiento de aplicación encuesta on-line

Estimados señores/as:

Estamos contactándolos por medio de correo e-mail para ver la posibilidad de que contesten una encuesta que es parte de una investigación desarrollada por el Departamento de Ecología de la Pontificia Universidad Católica de Chile. El objetivo principal de este estudio, es estimar el valor económico que para ustedes -que practican el buceo recreativo-, y sus experiencias recreacionales de buceo, poseen los niveles de biodiversidad que albergan las Áreas de Manejo y Extracción de Recursos Bentónicos (AMERBs) implementadas en sectores específicos de la costa de Chile. El conocimiento de sus percepciones y actitudes con relación a estos modelos de conservación en general, es también relevante para la temática principal del estudio.

La encuesta se aplica on-line, es anónima y tiene una duración aproximada de 20 minutos dentro de los cuales usted tendrá total libertad de dejar de responder en cualquier minuto. El encargado principal de la investigación, Dr. Stefan Gelcich, con mucho gusto contestará cualquier otra duda que usted pueda tener con relación a la temática de la investigación (sgelcich@bio.puc.cl, 02-3541914).

Si usted está de acuerdo en responderla por favor apriete el link con su consentimiento.

Estoy de Acuerdo ◯

No estoy de Acuerdo ◯

Opina sobre la biodiversidad marina 3

1. Información Personal

Esta encuesta es parte de un estudio del Departamento de Ecología de la Pontificia Universidad Católica, que busca indagar en las dimensiones humanas del manejo de recursos naturales marinos-costeros.

Necesitamos conocer tu opinión, buceador, sobre tu experiencia en el buceo, las condiciones ambientales de la costa central en Chile Continental y los distintos tipos de manejo que se llevan a cabo sobre ella con el propósito de proveer información que facilite y objetivice los procesos de toma de decisiones.

Por favor contesta a conciencia, tus respuestas serán de gran ayuda. Éstas serán tratadas con confidencialidad para fines académicos.

Además si te interesa, finalizado el período de ejecución de las encuestas te mandaremos un folleto informativo sobre reservas marinas mundiales.

1. Nombre (opcional)

2. Si te interesa recibir el folleto informativo sobre reservas marinas escribe tu dirección de correo electrónico.(opcional)

* 3. Edad?

* 4. Ciudad de Residencia?

* 5. Sexo

- ○ Femenino
- ○ Masculino

* 6. Ocupación

- ○ Estudiante
- ○ Dueño(a) de casa
- ○ Otro
- ○ Independiente/Empleado(a)
- ○ Jubilado

* 7. Nivel de Educación más alto alcanzado

- ○ Básica
- ○ Media
- ○ Pregrado
- ○ Postgrado
- ○ Técnico

Opina sobre la biodiversidad marina 3

2. Experiencia en el Buceo (1)

* 8. Cuánta experiencia tienes como buzo deportivo?

- Menos de un año
- Entre 5-9 años
- Más de 20 años
- Entre 1-4 años
- Entre 10-19 años

* 9. Cuál es tu principal motivación para practicar el buceo recreativo?

- Deporte/Contacto con la naturaleza
- Caza submarina
- Otro
- Búsqueda de aventura/retos personales
- Fotografía submarina

Otro (por favor especifique)

* 10. Cuál es el principal método de buceo que utilizas?

- apnea/snorkeling
- autónomo/skuba

* 11. Indica las 3 características que consideras más relevantes para que 1 sitio sea bueno para el buceo, siendo 1 la más importante y 3 la menos.

Que el agua sea cristalina y exista una buena visibilidad	
Que albergue una gran diversidad de flora y fauna marina	
Que tenga algún atractivo arqueológico/histórico/turístico	
Que tenga atractivos geomorfológicos (cuevas, paredes)	
Que de seguro observe mamíferos marinos en el buceo	

* 12. Eres miembro de algún grupo de buceo?

- Si
- No

Cuál?

* 13. Con qué frecuencia buceas durante el año?

- Muy esporádicamente (menos de 5 veces al año)
- Esporádicamente (entre 5 y 11 veces)
- Regular (entre 12 y 23 veces al año)
- Frecuentemente (entre 24 y 48 veces)
- Muy frecuentemente (más de 48 veces al año)

Opina sobre la biodiversidad marina 3

* 14. A cuál profundidad prefieres bucear?

- ◯ Hasta 15 metros
- ◯ 20 metros
- ◯ 30 metros
- ◯ 40 metros

* 15. Durante qué período del año buceas principalmente?

- ◯ Todo el año
- ◯ Primavera-Verano-Otoño
- ◯ Invierno
- ◯ Primavera-Verano
- ◯ Verano

Opina sobre la biodiversidad marina 3

3. Conservación y Actitudes Ambientales

* 16. Conoces alguna especie marina en peligro de extinción o en estado vulnerable?

- ◯ Si
- ◯ No

Si su respuesta es afirmativa porfavor mencione la primera que se le venga a la mente

* 17. Perteneces o has pertenecido a alguna organización de protección del medio ambiente?

- ◯ Sí
- ◯ No

Cuál

* 18. En general conoces/te informas sobre las especies que existen en los lugares que frecuentas en el buceo?

- ◯ Sí
- ◯ No
- ◯ Algunas veces
- ◯ Más o menos

* 19. Cuando vas a bucear a la costa continental de la zona central (desde Coquimbo hasta Pichilemu), cuáles son las 3 especies de peces que más te gusta observar? (nombre común o científico, en orden de preferencia).

1ª

2ª

3ª

* 20. En general, has notado un cambio en la abundancia de las especies de peces que más te gusta observar a lo largo de tu vida?

- ◯ Aumentaron mucho
- ◯ No cambiaron
- ◯ Decrecieron mucho
- ◯ Aumentaron poco
- ◯ Decrecieron poco

* 21. Dentro de la misma zona, cuales son las 3 especies de invertebrados (nombre común o científico) que más te gusta observar durante el buceo (siendo 1 tu preferida).

1ª

2ª

3ª

* 22. En general, has notado un cambio en la abundancia de las especies de invertebrados que más te gusta observar a lo largo de tu vida?

- ◯ Aumentaron mucho
- ◯ No cambiaron
- ◯ Decrecieron mucho
- ◯ Aumentaron poco
- ◯ Decrecieron poco

Opina sobre la biodiversidad marina 3

* 23. Si crees que la abundancia de fauna marina ha decrecido a que factor / factores lo atribuyes?

- [ ] Contaminación
- [ ] Cambio Climático
- [ ] Otro
- [ ] Sobrepesca
- [ ] Destrucción de habitat

Si contesto otro especifique porfavor

Opina sobre la biodiversidad marina 3

4. Reservas y Áreas Marinas (1)

* 24. Cree que es importante tener reservas marinas en Chile?

- ◯ Muy importante
- ◯ Importante
- ◯ Indiferente
- ◯ Medianamente importante
- ◯ Poco Importante

* 25. Conoce alguna reserva o área marina protegida en Chile?

- ◯ Sí
- ◯ No

Si la respuesta es sí, mencione la primera que se le viene a la mente

* 26. Qué función principal cree usted que cumplen las reservas marinas?

- ◯ Conservación
- ◯ Uso sustentable
- ◯ Educación
- ◯ Recreación

* 27. Has recbido información sobre reservas, parques o programas de protección marina desde que bucea?

- ◯ Muchas veces
- ◯ Bastantes veces
- ◯ Regular
- ◯ Muy ocasionalmente
- ◯ Nunca

* 28. Crees que el desarrollo de reservas marinas puedan contribuir en la mejora de los sitios de buceo?

- ◯ Muy de acuerdo
- ◯ De acuerdo
- ◯ No sé
- ◯ No

* 29. Sabes lo que es y como opera una área de manejo y explotación de recursos bentónicos(AMERB)?

- ◯ Sí
- ◯ No

* 30. Has buceado alguna vez dentro de una área de manejo de pescadores artesanales?

- ◯ Nunca
- ◯ Una vez
- ◯ Un par de veces
- ◯ Frecuentemente
- ◯ Muchísimas veces

* 31. Has tenido alguna vez problemas de ingreso a algún sitio para bucear?

- ◯ Si, muchas veces
- ◯ Si, pocas veces
- ◯ Una vez
- ◯ Nunca

Si quieres detallar tu experiencia, puedes hacerlo

Opina sobre la biodiversidad marina 3

* 32. Según tu experiencia como buceador, indica en qué medida estás de acuerdo o en contra con las siguientes afirmaciones? (N/A significa ni una de las anteriores)

	Muy de acuerdo	De acuerdo	Neutral	En contra	Muy en contra	N/A
Mientras más peces veo, es mejor el buceo	◯	◯	◯	◯	◯	◯
Normalmente me llevo a casa cosas que recolecto buceando	◯	◯	◯	◯	◯	◯
Frecuentemente buceo en los mismos sitios	◯	◯	◯	◯	◯	◯
Veo más especies en áreas de manejo que en áreas libres	◯	◯	◯	◯	◯	◯
Las mismas especies que veo en áreas libres, alcanzan mayor tamaño en áreas de manejo	◯	◯	◯	◯	◯	◯
Las mismas especies que veo en áreas libres, estan en mayor abundancia dentro de las áreas de manejo	◯	◯	◯	◯	◯	◯
Si pienso que un lugar no tiene especies, no buceo en él	◯	◯	◯	◯	◯	◯

* 33. Enumere de 1 a 5, en orden de importancia, qué factores priorizaría al momento de elegir una reserva marina en Chile, siendo 1 el factor más importante y 5 el menos importante a su juicio.

Belleza del lugar	▼
Abundancia de especies	▼
Presencia de especies emblemáticas	▼
Acceso a visitar	▼
Limpieza de las aguas	▼

* 34. Si hubiese una reserva marina en la zona central, la visitaría?

◯ Frecuentemente ◯ No sé ◯ Nunca

◯ Ocasionalmente ◯ Sólo en casos especiales ◯ Al menos una vez

* 35. En general crees que las áreas de manejo cumplen una función de conservación de la biodiversidad?

◯ Muy importante ◯ Importante ◯ No sé ◯ Normal ◯ Poco importante

* 36. Específicamente crees que la conservación de los recursos bentónicos (que viven en contacto con el fondo marino o en contacto con un sustrato, roca) es:

◯ Muy importante ◯ No sé ◯ Poco importante

◯ Importante ◯ Normal

Opina sobre la biodiversidad marina 3

* 37. Si se establecieran nuevas reservas marinas cuál cree usted que debiese ser la forma/formas de financiamiento? Porfavor marque todas las que usted considere necesarias.

- [ ] Impuestos (Estado)
- [ ] Entradas (Visitantes)
- [ ] Conseciones para el desarrollo de ecoturismo enfocado al buceo
- [ ] Contribución Voluntaria
- [ ] Financiamiento Extranjero
- [ ] Financiamiento Privado

* 38. Si las áreas de manejo, abiertas al buceo deportivo, contribuyeran al mantenimiento de la biodiversidad, crees que sería bueno que se otorgaran más concesiones de este tipo?

○ Sí ○ Sí probablemente ○ Neutral ○ No probablemente ○ No

5. Costos del Buceo

* 39. Cuántos kilómetros viajas desde tu casa hasta el sitio de buceo que más frecuentas en la zona central?

- ◯ Menos de 90
- ◯ Entre 90-130
- ◯ Entre 150-200
- ◯ Entre 200-400
- ◯ Más de 400

Indica el balneario que más frecuentas con fines de buceo (opcional)

* 40. Cuanto tiempo demora normalmente el viaje desde tu casa hasta el lugar de buceo?

- ◯ Menos de 1 hora
- ◯ 2 horas-2 horas y media
- ◯ Más de 3 horas
- ◯ 1 hora-1 hora y media
- ◯ 3 horas

* 41. Aproximadamente cuánto tiempo inviertes en cada ida a bucear?

- ◯ Medio día
- ◯ Todo un día
- ◯ Todo un fin de semana
- ◯ Más de 3 días

* 42. En función a tu experiencia buceando en Chile central continental, cuanto has gastado (en promedio) en los siguientes items en cada ida a bucear cuando el propósito de este viaje es exclusivamente realizar esta actividad

Locomoción

Alojamiento

Alimentación

Estacionamiento

Bote

Guía

Arriendo de equipo

Llenado de botellas

* 43. Cuál es el rango de ingresos de su hogar?

- ◯ No quiero contestar esta pregunta.
- ◯ $300.000-$500.000
- ◯ $3.500.000 o más
- ◯ $160.000 o menos
- ◯ $500.000-$1.200.000
- ◯ $160.000-$300.000
- ◯ $1.200.000-$3.500.000

Opina sobre la biodiversidad marina 3

6. Valorización del Buceo

Biólogos de la Pontificia Universidad Católica realizaron estudios que comparaban la biodiversidad existente dentro y fuera de Áreas de Manejo y Explotación de Recursos Bentónicos en El Quisco y Algarrobo. Los resultados indicaron que además de haber una mayor abundancia de las especies manejadas con fines comerciales (locos, lapas, erizos, y otros) también existía mayor diversidad y abundancia de peces e invertebrados, como por ejemplo lo muestra la siguiente figura. (Ver abajo)

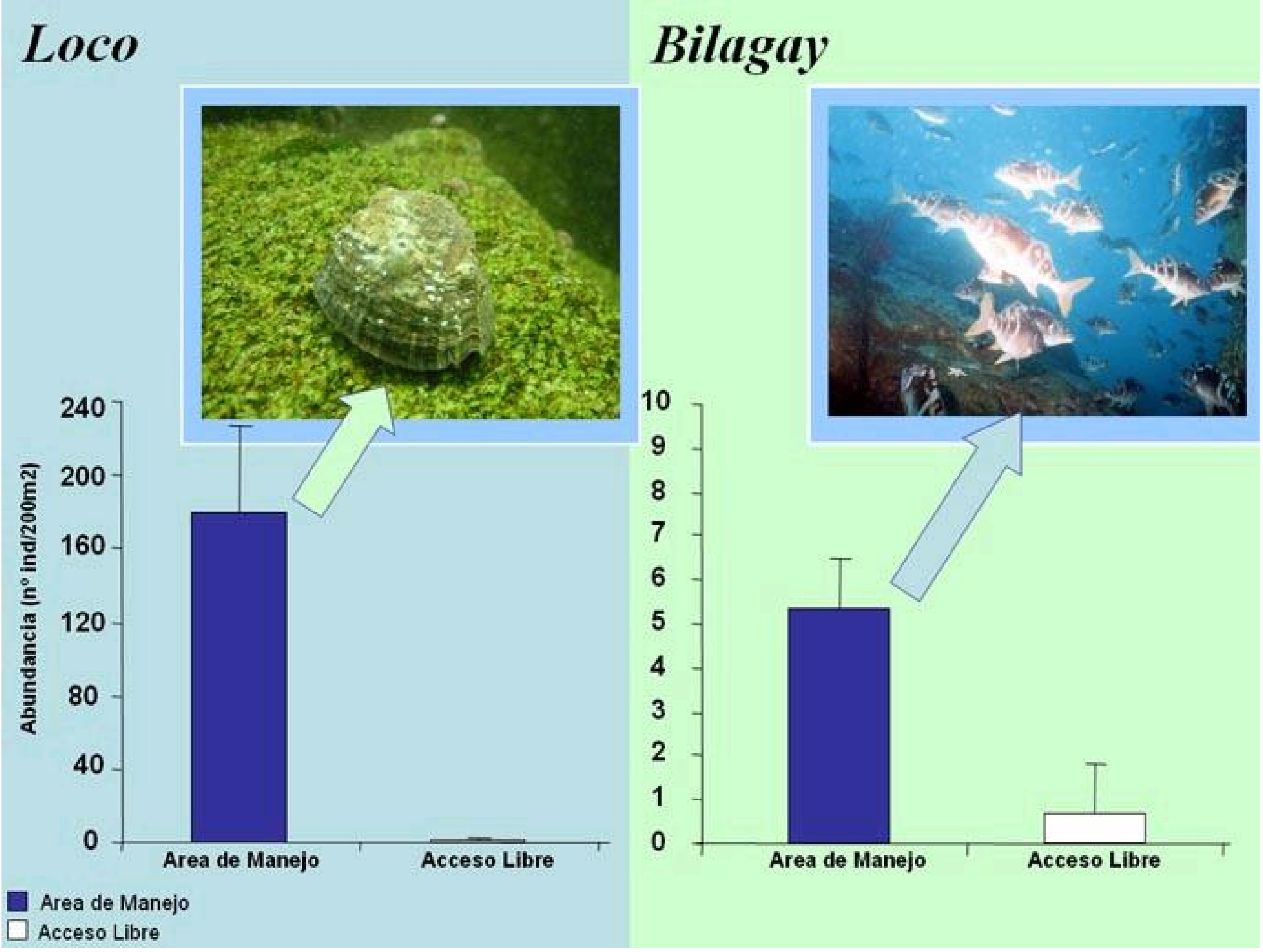

* 44. Si pudiera acceder a estos sitios de mayor biodiversidad y mejor calidad ambiental, en cuanto estimas que aumentarían tus viajes a bucear durante el año?

- ◯ 0
- ◯ Aprox 5 veces más
- ◯ Aprox 12 veces más
- ◯ Aprox 24 veces más
- ◯ Otra cantidad

Si dijo otra cantidad porfavor especifique

____

Las siguientes preguntas plantean un escenario hipotético que te solicitamos responder como si se tratara de una situación real.

Opina sobre la biodiversidad marina 3

* 45. Si los pescadores artesanales del Quisco y Algarrobo decidieran abrir al público sus áreas de manejo (donde se llevaron a cabo los estudios) , estarías dispuesto/a a pagar una entrada de $x por bucear en este sitio?

- Si
- No
- Creo que se deberia pagar de otra forma, no una entrada

* 46. Por qué estás/no estás de acuerdo con pagar una entrada de $x?

- Si estoy de acuerdo porque valoro el hecho de observar mayor biodiversidad cuando buceo.
- Si estoy de acuerdo porque incentivaría económicamente a los pescadores a conservar la biodiversidad y su forma de vida tradicional.
- No estoy de acuerdo en pagar por bucear,aunque las áreas sean cuidadas por los pescadores, el mar es de todos los chilenos.
- No valoro las diferencias de biodiversidad que los estudios comprueban que existen dentro y fuera de las áreas de manejo.

* 47. Cuánto estaria usted dispuesto a pagar por bucear en estos sitios de mayor biodiversidad?

- $3.000
- $5.000
- $8.000
- $10.000
- $15.000
- Otro valor
- No estoy de acuerdo con pagar

Otro valor (por favor especifique))

* 48. Como cree usted que se deberia financiar?

- No debería ser financiado
- Entrada
- Impuestos
- Subvención a los pescadores
- Aporte voluntario

Opina sobre la biodiversidad marina 3

* 49. Las áreas de manejo no sólo permiten el uso sustentable de las especies con fines comerciales , además permiten un aumento en la cantidad de especies no explotadas, lo que implica un aporte indirecto para la conservación de la biodiversidad.

Cómo cree usted que debiera ser retribuido este beneficio?(Conteste la opción que más lo identifique.)

- ◯ No creo que debiera ser retribuido
- ◯ Mediante una bonificación estatal en beneficio de las áreas de manejo
- ◯ Pagando entradas para bucear en esas zonas
- ◯ Con contribuciones voluntarias a un fondo solidario
- ◯ Otorgando facilidades a los pescadores que cuidan de la áreas en términos de pago de impuestos, patentes, etc.

Si quieres detallar tu punto de vista por favor adelante

Muchas gracias por tu tiempo e interés.
Si te interesa saber más sobre conservación marina te recomiendo los sitios www.ecim.cl, www.directemar.cl o www.conama.cl/gefmarino.
